# Critical evaluation of cancer risks in glomerular disease

**DOI:** 10.1016/j.tranon.2022.101376

**Published:** 2022-02-24

**Authors:** Zaw Thet, Alfred K. Lam, Dwarakanathan Ranganathan, Soe Yu Aung, Thin Han, Tien K. Khoo

**Affiliations:** a School of Medicine and Dentistry, Menzies Health Institute Queensland, Griffith University, Gold Coast, Queensland 4222, Australia; b Department of Nephrology, Central Queensland Hospital and Health Services, Rockampton, Queensland 4700, Australia; c Faculty of Medicine, University of Queensland, Herston, Queensland 4006, Australia; d Pathology Queensland, Gold Coast University Hospital, Southport, Queensland 4215, Australia; e Department of Nephrology, Metro North Hospital and Health Services, Herston, Queensland 4006, Australia; f Department of Oncology, Central Queensland Hospital and Health Services, Rockhampton, Queensland 4700, Australia; g School of Medicine, University of Wollongong, Keiraville, New South Wales 2522, Australia

**Keywords:** Cancers, Malignancy, Glomerular disease, Glomerulonephritis, Nephrotic syndrome

## Abstract

The increased cancer incidence in patients with glomerular disease can be secondary to an intrinsic immune dysfunction associated with the disease or/and extrinsic factors, especially immunosuppressants. The treatment for paraneoplastic glomerulopathy is different from primary glomerular disease. Immunosuppressive therapy often used for primary glomerulopathy may aggravate concomitant cancers in patients with paraneoplastic glomerulopathy. In membranous nephropathy (MN), measurement of serum circulating autoantibodies against podocyte transmembrane glycoprotein M-type phospholipase A2 receptor (PLA2R) and thrombospondin type 1 domain-containing 7A (THSD7A), immunohistochemical staining of kidney tissue for glomerular PLA2R, THSD7A, neural epidermal growth factor-like 1 protein (NELL-1) and specific types of immunoglobulin G (IgG) may be useful adjuncts when screening for underlying malignancies. This review addresses overall cancer risks in individuals with glomerular diseases and employment of biomarkers available for MN. We propose a scheme of screening of cancers frequently reported in the setting of glomerular disease.

## Highlights

• The increased cancer incidence in patients with glomerular disease can be secondary to an intrinsic immune dysfunction associated with the disease or/and extrinsic factors, especially immunosuppressants.
• Paraneoplastic glomerulopathy is sometimes misdiagnosed as primary glomerulopathy.
• The treatment for paraneoplastic glomerulopathy is different from primary glomerular disease.
• In membranous nephropathy, serum circulating autoantibodies against PLA2R and THSD7A, immunohistochemical tissue markers for glomerular PLA2R, THSD7A and specific types of immunoglobulin G (IgG) may be used for identifying underlying malignancies.
• A scheme of screening of cancers frequently reported in the setting of glomerular disease is important.

## Graphical abstract

**Figure:**
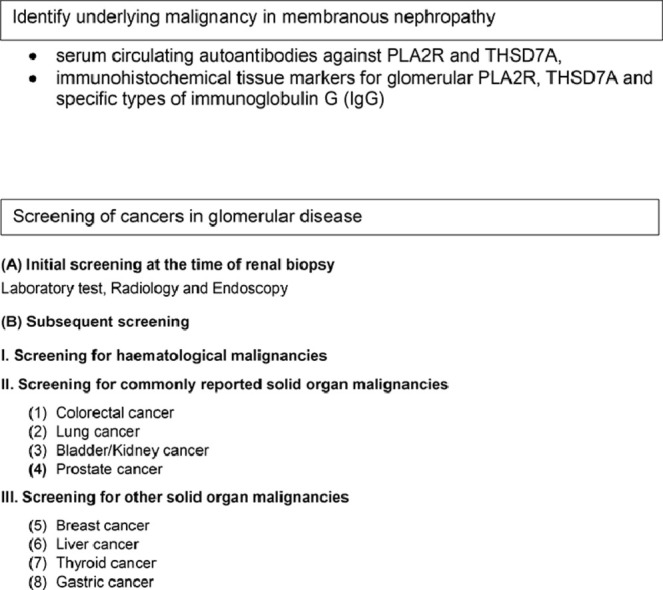

## Introduction

Glomerular disease is a leading cause of end stage kidney disease, and it can be idiopathic or result from inherited or acquired disorders, occurring in the setting of systemic autoimmune disease, infections, drugs, or malignancy [1], [2], [3], [4], [5], [6], [7]. Glomerular diseases can be classified by glomerular cell target, mechanism of injury or presence (or absence) of systemic involvement and by clinical entities, such as glomerulonephritis, nephrotic syndrome, and asymptomatic haematuria and proteinuria. However, there may be considerable overlap in clinical presentation.

Observational studies, case reports and case series have supported a link between glomerular disease and cancer. Some glomerular diseases require treatment with long-term immunosuppressants known to have oncogenic adverse effects and they may accelerate oncogenesis and neoplastic progression by direct mutagenesis or disruption in immune surveillance [8], [9], [10], [11], [12]. In addition, glomerular diseases associated with malignancy (paraneoplastic glomerulopathy) may be secondary complications of primary cancers and due to altered immune responses associated with cancer [13], [14], [15], [16]. On the other hand, long-term use of immunosuppressants, exposure to environmental factors, and oncogenic viral pathogens, such as hepatitis B virus (HBV), hepatitis C virus (HCV), Epstein Barr virus (EBV), human papilloma virus (HPV) could play important roles in the development of de novo cancers [15,[18], [19], [20], [21], [22], [23], [24], [25], [26], [27]]. Furthermore, genetic predisposition and lifestyle-related risk factors (for example, smoking, exposure to ultraviolet radiation) may increase cancer risk in patients with glomerular disease [11,27].

The pathogenesis of glomerular disease secondary to malignancy is poorly understood [13], [14], [15], [16]. Findings of remission in glomerular disease after ablation of cancer by surgery or anticancer drugs as well as relapse of glomerular disease after recurrence of neoplasia suggests the occurrence of paraneoplastic disease [15]. The proposed pathogenic mechanisms include: (i) appearance of autoantibodies against a tumour antigen with analogous immunological properties to those of an antigen residing within a component of the glomerulus, which result in in-situ immune complex production; (ii) production and trapping of circulating immune complexes in glomerular capillaries, (iii) reaction of circulating antibodies with tumour antigens which are deposited in the glomerular membrane (iv) involvement of external factors such as oncogenic viruses [13], [14], [15].

Intrinsic immune dysfunction associated with glomerular disease may increase the risk of cancer [[17], [18], [19], [20], [21],^26^]. Animal studies indicated that T-helper 2 polarization has an important role in the development of thymoma-associated glomerular lesions. In minimal change disease and focal segmental glomerulosclerosis, overexpression of interleukin (IL)-13, a T-helper 2 cytokine, was noted to induce the disease [18,19] but how this overexpression is linked to malignancy is unknown. In systemic lupus erythematosus, increased interleukin-6 (IL-6) activity, overstimulation of T- and B-cells, coupled with defects in the immune system's surveillance system may increase the risk of cancer [20,21]. In membranous nephropathy, many antigens in podocytes associated with circulating antibodies responsible for pathogenesis of the disease have been identified and some markers of use in identifying malignancy related MN [26].

The risk of developing a de novo cancer is significantly associated with immunosuppressant use and cumulative exposure [36,37]. In paraneoplastic glomerulopathy, glomerular disease can precede the identification of an underlying malignancy, be diagnosed synchronously at the time of kidney biopsy or after biopsy proven glomerular disease [36,39]. Paraneoplastic glomerulopathy can be misdiagnosed as primary glomerular disease, thus potentially leading to the introduction of immunosuppressive therapy that may be harmful and may induce more rapid tumour growth.

The aim of this review is to determine the risk of paraneoplastic tumours and de novo cancers in patients with glomerular disease and assess recommendations in cancer surveillance. In addition, this review focus on membranous nephropathy which is used as a prototype in understanding the association of glomerular disease and cancer. Several advances have been made in the pathophysiology of membranous nephropathy and the employment of biomarkers may provide more precise differentiation between idiopathic and malignancy-associated nephropathy [28], [29], [30], [31].

## Cancer risk in glomerular disease

A population-based study which extracted data from the Danish Kidney Biopsy Registry with a study duration of 30 years (1985-2014) and follow-up period of 6.4 years (± 3.7 years) found that 16% (n=911) of 5,594 patients with glomerular diseases had cancer (IRR=1.8, 95% CI = 1.40-2.10) [35,36]. The incidence of cancer was 0.5%/year in patients <45 years of age whereas the risk in those aged 45-64 years and > 64 years was 2.7%/year and 5.6%/year, respectively. The increased incidence of cancer was mainly limited to a 1-year duration before and after biopsy, however, skin cancer risk increased over time. The overall cancer incidence increased 3–10 years after biopsy, which may be related to cumulative immunosuppressant exposure. Malignancies with significantly increased rates include lung, prostate, kidney, non-melanoma skin cancer, non-Hodgkin's lymphoma, myeloma, and leukaemia.

Based on the Danish Kidney Biopsy Registry, there was an increase in cancer risk in nephrotic syndrome patients without a prior history of cancer [34]. A registry-based study that involved 4,293 patients with nephrotic syndrome reported the 5-year risk of any cancer was 4.7% (SIR= 1.73, 95% CI =3.68-5.42). Of these, 7.9% (*n*=338) developed cancer during a median follow-up of 5.7 years. Approximately one third of cancers were diagnosed within one year of nephrotic syndrome onset. The association of cancer and glomerular disease peaked within a 6-month period following the diagnosis of nephrotic syndrome (SIR=6.84, 95% CI =5.48-8.42) and subsequently reduced at 6-12 months (SIR=1.79, 95% CI =1.09-2.76) and after a year (SIR=1.34, 95% =1.17-1.53). The association was most pronounced in younger patients (SIR=2.82, 95% CI =1.88-4.08 for age group 0-29 years, SIR=1.52, 95% CI =1.14-1.97 for age group 30-49 years, SIR=1.84, 95% CI =1.58-2.14 for age group 50–69 years, SIR=1.47, 95% CI =1.16-1.83 for age group ≥70 years, respectively). There was a significant association of nephrotic syndrome and haematological malignancies which included multiple myeloma (SIR=19.2, 95% CI =13.8-26.0), Hodgkin's lymphoma (SIR=11.3, 95% CI =5.41-20.8), non-Hodgkin's lymphoma (SIR=3.16, 95% CI =1.90-4.93). In addition, other solid cancers such as renal cancer (SIR=2.67, 95% CI =1.33-4.78) and lung cancer (SIR=2.06, 95% CI =1.57-2.66) were noted.

A single-centre retrospective study with 822 patients with primary or secondary glomerulonephritis in Korea found that 5.5% (n=45) of patients developed de novo cancer ≥ 6 months after kidney biopsy during a mean follow-up period of 58.9 ± 44.5 months [37]. This study is the first to report on cancer risks in an Asian population with glomerulonephritis. In this study, patients who had a previous diagnosis of cancer within 6 months after kidney biopsy were excluded. The most common malignancies detected were hepatocellular carcinoma (13.3%), colon carcinoma (11.1%), papillary thyroid carcinoma (8.9%), gastric carcinoma (6.7%), prostate carcinoma (6.7%), and lymphoma (6.7%).

### Membranous nephropathy (MN) as a prototype in understanding the association of glomerular disease and cancer

MN is the most common cause of nephrotic syndrome in Caucasian adults [22]. In MN, approximately 70% of cases are considered as primary and 30% are due to secondary causes such as malignancy, chronic infection, drugs, systemic lupus erythematosus, sarcoidosis and other autoimmune diseases [22,23]. MN is frequently used as a model to better understand the association of glomerular disease with cancer, although such association also occurs in other glomerular diseases such as minimal change disease, IgA nephropathy, ANCA-positive and ANCA-negative crescentic glomerulonephritis, MPGN, fibrillary glomerulonephritis, thrombotic microangiopathy [17,38,39]. MN has received considerable attention due to recent advances in understanding the pathogenesis of disease and availability of biomarkers in differentiation between primary and secondary MN [22,23].

Current literature suggests that MN is closely associated with solid cancers and haematological malignancies [28,32,35,36,41,42]. In a Norwegian-based registry study with 161 MN patients, the SIR of cancer was 2.25 and the annual incidence continued to increase after 5 years from biopsy-proven diagnosis of nephropathy, compared with age- and sex-adjusted general population. Older age and heavy smoking (>20 pack years) were strong predictors of cancer in these patients [32].

A meta-analysis (6 observational studies, 785 patients) reported a 10% prevalence of cancer in MN [41]. The mean age of patients with MN who had cancer was 66.35 ± 6.75 years. The most common cancer type noted in these patients was lung cancer (26%) followed by prostate cancer (15%) and haematological malignancy (14%). Many cancers were diagnosed at the time of or after the diagnosis of MN. The diagnosis of cancer preceded the diagnosis of MN in 20 ± 6.8% of the cancers. Bjørneklett et al found 33 out of 161 Norwegian patients developed malignancy over a mean duration of follow-up of 6.2 years and 80% of malignancy was detected with the first 12 months of diagnosis of MN [32]. The median time from diagnosis of MN to diagnosis of cancer was 60 months (range, 0-157 months) whereas the mean annual incidences of cancer for 0-5 years and >5 to 15 years from diagnosis of glomerular disease were 2.1/100 person-years and 2.8/100 person-years, respectively. Lefaucheur et al reported that 52% of French patients with cancer-associated MN were undiagnosed with cancer at the time of kidney biopsy but had cancer-related symptoms [33]. Zhang et al revealed that Chinese patients with MN were significantly older, had higher serum creatinine and a lower estimated glomerular filtration rate (eGFR) than idiopathic MN patients [28]. Another study from China found similar findings and noted that patients with malignancy associated with MN were older (64.4 ± 8.7 vs. 51.6 ± 11.1 years, *p* = 0.003), had lower serum albumin levels (22.4 ± 5.8 vs. 26.7 ± 5.0 g/L, *p* = 0.034) and higher serum C-reactive protein (CRP) (11.2 ± 9.3 vs. 2.8 ± 4.9 mg/L, *p* = 0.003) when compared to those without malignancy [42].

There is limited evidence on the prognostic difference between individuals with MN with de novo cancer or those with paraneoplastic glomerulopathy. Regardless of the latter, concurrent MN and cancer is associated with poor kidney and patient survival. In a cohort study with record linkage between the Norwegian Kidney Biopsy Registry and Norwegian Cancer Registry, patients with cancer and MN had a greater mortality rate than patients with MN and without cancer over a mean follow-up period of 6.2 years (67% vs. 26%, *p* < 0.001)[32]. A higher mortality rate difference (67% vs. 8.3%, *p* < 0.001) was reported in a French retrospective study that involved 240 patients with membranous nephropathy [33]. In this study, 10% of patients had malignancy and 44% of mortality was due to neoplasia.

The employment of novel biomarkers can be helpful in distinguishing between idiopathic MN and secondary MN including malignancy-related MN (see Table 2). Transmembrane glycoprotein M-type phospholipase A2 receptor is an autoantigen present in the podocyte and is the major target antigen in primary MN [28,29,78]. Circulating autoantibodies to podocyte transmembrane glycoprotein M-type phospholipase A2 receptor (anti-PLA2R) has been regarded as a biomarker for primary MN [28,[43], [44], [45], [46], [47]]. It is positive in up to more than 80% of cases of primary MN [17,28,40,46,[48], [49], [50], [51]] but varies in prevalence from 0 to 64% in secondary MN of any cause and up to 30% in malignancy related membranous nephropathy [49]. Glomerular PLA2R antigen deposition was detected by immunoblot or immunofluorescence assay [52,[54], [55], [56],^63^,^67^] and showed a good correlation with serological tests of anti-PLA2R [72]. A meta-analysis of the diagnostic accuracy of serum anti-PLA2R as well as glomerular PLA2R antigen in primary MN [63], was summarised in Table 3. Due to the limited sensitivity of PLA2R serological tests, its utility in malignancy-related membranous nephropathy requires further consideration and should be employed as a supportive rather than diagnostic test.

Recent studies have shown that antibodies against another podocyte antigen thrombospondin type 1 domain-containing 7A (THSD7A) was detected in approximately 5-12% of patients with membranous nephrology who are PLA2R-negative [[57], [58], [59], [60],^73^]. Glomerular THSD7A staining was correlated strongly with the serum anti-THSD7A antibody testing [64]. A high incidence of cancer was reported in patients with THSD7A-associated MN and it was predominantly expressed in PLA2R negative cases [57,61,62,[64], [65], [66]]. A systematic review (10 studies involving 4,121 patients with MN) reported that the prevalence of THSD7A was 3% in all patients and 10% in PLA2R-negative patients [30]. Among THSD7A-postive patients, the incidence of malignancy was between 6% and 25%. Recently, a meta-analysis (14 studies involving 4,545 patients with MN) revealed the accuracy of THSD7A antibody in the diagnosis of primary MN [77] (Table 2).

Dual positivity of PLA2R and THSD7A was detected in 10% of patients with primary MN [73]. It is worth noting that PLA2R and THSD7A are used to differentiate primary MN from secondary MN. In addition, the predictive value of negative PLA2R and positive THSD7A results may be useful when screening for underlying malignancies in MN.

Glomerular IgG4 deposition was identified predominantly in primary MN whereas predominant depositions of IgG1/IgG2/IgG3 were often seen in secondary MN including malignancy-related MN.[40,47,68,[74], [75], [76]] In patients with MN, the absence of glomerular IgG4 deposition was an independent predictor for the development of malignancy (HR =0.07, 95% CI =0.01–0.57, *p*=0.014)[42]. Inflammatory cells were rare in primary MN and the presence of >8 inflammatory cells per glomeruli on kidney biopsy was observed in a study on cancer-related membranous glomerulonephritis but this finding was not replicated in other studies [33].

A major technological leap involving laser microdissection of glomeruli followed by mass spectrometry has identified additional antigens which may serve as biomarkers and may identify underlying causes of MN [70]. Neural epidermal growth factor-like 1 protein (NELL-1) is a potential new biomarker in the diagnosis of malignancy associated MN [70], [71]. Although technology allows identification of target antigens on podocytes, the molecular mechanisms of glomerular damage after antibody binding to target podocyte antigen as well as the pathophysiological mechanisms between glomerular disease and cancer are not entirely understood. Recently, Bobart et al proposed a new classification of MN based on correlating clinical features, biochemistry, pathology, follow-up data including seven septuple which consists of target antigens, namely PLA2R, THSD7A, Semaphorin 3B (SEMA3B), NELL-1, Protocadherin 7 (PCDH7), Exostosin 1/Exostosin 2 (EXT1/EXT2) and Neural cell adhesion molecule 1 (NCAM-1) [82]. Only Nell-1 positive and seven septuple negative status seems to be associated with malignancy. This approach of differentiating between primary and secondary MN is currently available in a few specialized centres globally. Collaborative studies are required to improve our insight into application of target antigen-specific classification system for membranous nephropathy Table 1.

**Table 1:** Characteristics of paraneoplastic tumours and de novo cancers in glomerular disease.

Pathogenesis Remains poorly understood
Incidence (all types of glomerular disease) (in Europeans) [8,36,78] 3.2%-16% (both paraneoplastic glomerulopathy and de novo cancer) Incidence (all types of glomerular disease) (in Asians) [37] 5.5% (de novo cancer 6 months after renal biopsy) Incidence (nephrotic syndrome) (in Europeans) [34] 7.9% (both paraneoplastic glomerulopathy and de novo cancer)
Onset Paraneoplastic glomerulopathy should be considered as a likely diagnosis when cancer is diagnosed at the time of renal biopsy or 6-12 months before/after biopsy proven glomerular disease. De novo cancer is more likely if neoplasia develops 12 months after renal biopsy proven glomerular disease.
Risk factors Intrinsic: Immune dysfunction/Genetics Extrinsic: Immunosuppressants, Infections, Environmental factors such as smoking, ultra-violet radiation.
Risk of both paraneoplastic tumour and de novo cancer by type of glomerular disease (in Europeans) [36] • Unclassified glomerular disease (IRR=4.9, 95% CI = 3.90-6.10) • MPGN (IRR=3.1, 95% CI =1.90-4.70) • Endocapillary glomerular disease (IRR=3.0, 95% CI =1.20-6.20) • Proliferative glomerular disease (IRR=2.9, 95% CI =1.20-6.00) • Minimal change disease (IRR=2.4, 95% CI =1.40-3.70) • Focal segmental glomerular sclerosis (IRR=2.4, 95% CI =1.60-3.50) • Mesangioproliferative glomerular disease (IRR=1.8, 95% CI =1.20-2.50) • ANCA associated vasculitis (IRR=1.8, 95% CI =1.10-2.50) • Lupus nephritis (IRR=1.7, 95% CI =1.50-1.80) • Membranous glomerular disease (IRR=1.5, 95% CI =1.10-2.10)
Risk of de novo cancer by type of glomerular disease within 3 years after renal biopsy (in Europeans) [36] • Membranoproliferative glomerulonephritis: 20% (ag >64 years) vs 9% (age 45-64 years) • Membranous nephropathy: 18% vs 7% • Minimal change disease: 17% vs 7% • Focal segmental glomerulosclerosis: 16% vs 5% • Mesangioproliferative glomerular disease: 13% vs 4% • Endocapillary glomerular disease: 12% vs 9% Risk of de novo cancer by type of glomerular disease with (SIR) (in Asians) [37] • Lupus nephritis (standardised incidence ratio (SIR)= 39.46; 95% CI =7.93-129.29) • Crescentic glomerulonephritis (SIR= 10.06; 95% CI =1.13-43.68) • Membranous nephropathy (SIR= 8.92; 95% CI =4.44-16.30) • Focal segmental glomerulosclerosis (SIR= 7.73; 95% CI =2.49-19.17) • IgA nephropathy (SIR= 6.05; 95% CI =3.12-10.77)
Commonly reported solid cancers and haematological malignancies (all types of glomerular disease) • In Europeans (both paraneoplastic glomerulopathy and de novo cancer); Lung carcinoma, prostate carcinoma, renal carcinoma, non-Hodgkin's lymphoma, myeloma, and leukaemia [36] ⁎⁎ • In Asians (de novo cancer); Hepatocellular carcinoma, colon carcinoma, papillary thyroid carcinoma, gastric carcinoma, prostate carcinoma, and lymphoma37**
Commonly reported solid cancers by types of paraneoplastic glomerulopathy (in order of increasing frequency) [39] • Membranous nephropathy: Lung, stomach/oesophagus, renal, prostate, thymus, colon/rectum, breast, pancreas, sarcoma, bladder, larynx/pharynx, liver carcinomas, and brain tumour⁎⁎ • Minimal change disease: Thymus, lung, colon/rectum, renal, bladder, breast, pancreas, stomach/oesophagus carcinomas, sarcoma, and ovary carcinoma⁎⁎ • Crescentic glomerulonephritis: Renal, stomach/oesophagus, lung, prostate, thymus, breast, larynx/pharynx, colon/rectum, liver, bladder carcinomas, sarcoma, and ovary carcinoma** • IgA nephropathy: Renal, lung, and stomach/oesophagus carcinomas⁎⁎ • Mesangioproliferative glomerular disease: Lung, renal, breast, stomach/oesophagus, prostate, bladder, thymus, and ovary carcinomas⁎⁎ • Focal segmental glomerulosclerosis: Renal, thymus, lung, pancreas, breast carcinomas, sarcoma, and stomach/oesophagus carcinoma⁎⁎

⁎⁎ listed from highest to lowest reported frequency.

**Table 2:** Diagnostic accuracy of biomarkers in primary membranous nephropathy (MN) [63,77].

Serum anti-PLA2R autoantibody	
Sensitivity	65% (95% CI =63-67)
Specificity	97% (95% CI =97-98)
Positive likelihood ratio	15.65 (95% CI =9.95-24.62)
Negative likelihood ratio	0.37 (95% CI =0.32-0.42)
Diagnostic odds ratio	50.41 (95% CI =31.56-80.52)
Glomerular PLA2R antigen	
Sensitivity	79% (95% CI =76-81)
Specificity	90% (95% CI =88-92)
Positive likelihood ratio	8.17 (95% CI =5.60-11.93)
Negative likelihood ratio	0.25 (95% CI =0.19-0.33)
Diagnostic odds ratio	39.37 (95% CI =22.18-60.13)
Serum anti-THSD7A autoantibody	
Sensitivity	4% (95% CI =2-7)
Specificity	99% (95% CI =98-100)
Positive likelihood ratio	5.40 (95% CI =2.40-11.90)
Negative likelihood ratio	0.97 (95% CI =0.95-0.99)
Diagnostic odds ratio	6.00 (95% CI =31.56-80.52)
Useful adjuncts when screening for underlying malignancies	
Malignancy related membranous nephropathy may be highly likely in the following: • negative serum anti-PLA2R antibody/glomerular PLA2R antigen • positive serum anti-THSD7A antibody/glomerular THSD7A antigen • predominant IgG1/IgG2/IgG3 glomerular deposition	

## Cancer screening in glomerular disease

Currently, there is no consensus among clinicians with regards to cancer screening in patients with glomerular disease. In a single-centre study from Italy, full screening {tumour biomarkers (Ca125, CEA, Ca19.9, Ca15.3, alpha fetoprotein, prostate specific antigen), stool for occult blood, chest x-ray, abdominal ultrasound, gastroscopy, total body computed tomography (CT), colonoscopy, mammography, cervical smears and urine cytology} for cancer was performed. In this study, 7.4% (n=12) of malignancies were diagnosed among 163 patients with biopsy proven glomerulonephritis and nephrotic syndrome during a median follow-up of 5.7 years (range, 1 month-21 years) [8].

In general, it is a standard practice to screen for malignancy especially in older patients with newly diagnosed glomerular disease. The Danish Renal Biopsy Registry found the incidence of cancer was rare in patients < 45 years with glomerular diseases but increased significantly beyond 45 years of age [36]. In patients with ANCA associated vasculitis, it was proposed that cancer screening should be based on the clinical context and supportive screening criteria, such as age ≥ 45 years, heavy current or ex-smokers, heavy alcohol users and patients with a history of previous cancer, concurrent hepatitis (HBV, HCV), human immunodeficiency virus (HIV) infections and significant family history of malignancy [11]. A further emphasis was mentioned for patients who had relapsing, or refractory glomerular disease treated with additional immunosuppressants that incurred a higher cumulative dose and longer duration as well as those who received a cumulative dose of >36g of cyclophosphamide. Pani et al recommended screening for cancer every 5 years for younger patients (<50–60 years) and every 3 years in elderly patients (>60 years) [8]. The screening approach is comprehensive, and it includes three levels of physical examination and investigation tests. First level assessment included skin examination, breast/testicular examination, chest x-ray, abdominal and cervical ultrasound, faecal occult blood test (if positive, to proceed to colonoscopy/gastroscopy). If first level assessment was normal, the second level of approach {mammography and gynaecological review, cystoscopy if there is haematuria; serum prostatic-specific antigen and rectal exam (prostate biopsy if neoplasia is suspected)} was recommended. In high-risk patients (> 60 years, smokers, alcoholic; thromboembolism; HIV/HBV/HCV infection; prolonged immunosuppressive treatment; negative anti-PLA2R antibodies in MN), a further third level of investigations (CT scan, colonoscopy, endoscopy) should be performed if second level assessment was unremarkable.

Preliminary findings suggested that 18F-fluorodeoxyglucose positron emission tomography/computed tomography (FDG-PET) may be useful in detecting cancer in patients with MN [79]. To date, utilisation of FDG-PET is not routinely adopted in screening for malignancy in patients with glomerular diseases. Although there were studies evaluating the cost-effectiveness as well as the benefit-to-harm ratio of a comprehensive cancer screening in the general population, there was no such study in patients with glomerular disease [80].

The Kidney Disease Improving Global Outcomes (KDIGO) guidelines recommended cancer screening in patients with MN though the guidelines did not detail how and when it should be performed [9,10]. It is also not known whether the guidelines are applicable to other types of glomerular disease. In a recent KDIGO initiated conference, a global multidisciplinary panel of clinicians and scientists discussed controversies in onco-nephrology [81]. However, interaction between cancer and glomerular disease was not covered in this conference. With regards to haematological malignancies, we agree with Pani et al that full screening is warranted in patients with unexplained anaemia, monoclonal protein spike (paraprotein) on serum electrophoresis, hepatomegaly, splenomegaly, enlarged lymph nodes, night sweats, fever, and weight loss [8]. Screening should include bone marrow biopsy, total body CT and/ or FDG-PET scan. Regarding screening for solid cancers, the utility of FDG-PET imaging requires further study.

A high degree of clinical suspicion for underlying cancer should be maintained and appropriate cancer screening be considered in patients with glomerular disease. This is because delay in diagnosis and introduction of immunosuppressive therapy may be harmful and induce rapid progression of an occult malignancy. It is important that patients with glomerular disease are compliant with standard of care cancer screening programs recommended in the general population. Though recommendations on cancer screening in the general population may vary around the world, we support an approach that adapts consensus cancer screening recommendations from medical societies in America with screening of solid cancers in general population described in Table 3. This approach should be adopted based on the clinical context after explaining the benefits and harms of cancer screenings.

**Table 3:** Cancer screening in patients with glomerular disease [8,11,50,53].

(A) Initial screening at the time of renal biopsy*Assess for solid cancer and haematological malignancy that may be associated with glomerular disease (i.e., paraneoplastic glomerulopathy) • Laboratory tests: Full blood examination, renal function tests, liver function tests, lactate dehydrogenase Tumour markers where appropriate Bone marrow biopsy when haematological malignancy is suspected • Radiology: CT chest, abdomen, and pelvis with contrast (Consider FDG-PET CT scan if renal function is poor) • Endoscopy: Gastroscopy and colonoscopy where necessary (B) Subsequent screening*Assess for concurrent de novo cancer.Make sure patients are compliant with gold-standard cancer screening programs recommended for general population if readily accessible.Depending on pre-existing high malignancy risk factors and intensity and duration of immunosuppressive treatment, more frequent screening may be considered.I. Screening for haematological malignancies a Look for causes of anaemia, hepatomegaly, splenomegaly if present. b Consider serum electrophoresis, bone marrow biopsy, total body CT scan or positron emission tomography scan in case of unexplained anaemia, hepatomegaly, or splenomegaly, enlarged lymph nodes, night sweats, fever, and weight loss II. Screening for commonly reported solid organ malignancies (1) Colorectal cancer# * a. Adult age 50-75 years* who are at average risk for colorectal cancer should been screened. b. Patients should consult with their health care providers to choose the test that is right for them. Consider faecal occult blood test (FOBT) annually or colonoscopy when clinically indicated. (1) Lung cancer# * a. Consider low dose computed tomography for adults age 55-79 years* who are at high risk for lung cancer (current smoker or who have quit within 15 years with 30 pack year smoking history). (1) Bladder/Kidney cancer* a. Annual urine examination for red cell morphology and malignant cells especially in patients who received cumulative dose >36g of cyclophosphamide or those who receive additional immunosuppressants for recurrence of glomerular disease. b. Imaging and cystoscopy if unexpected monomorphic haematuria or positive urine cytology (1) Prostate cancer# * a. Men age 55-69 years* who are at average risk for prostate cancer talk to a clinician about the benefits and potential harms of prostate specific antigen (PSA) testing before deciding if screening is right for them. III. Screening for other solid organ malignancies (1) Breast cancer# *: a. Yearly or second yearly mammogram in women age 50-74 years*. (1) Liver cancer* a. Liver ultrasound and serum alpha-fetoprotein in patients with chronic hepatitis B or C. (1) Thyroid cancer* a. No standard or routine screening tests used for early detection of thyroid cancer. (1) Gastric cancer* a. In countries with a low incidence of gastric cancer, routinely screening is not done to detect early gastric cancer +. b. In countries with a high incidence of gastric cancer, screening of gastric cancer by upper endoscopy or contrast radiography is practiced +. c. Screening for gastric cancer is recommended in individuals at increased risk of gastric cancer include gastric adenomas, pernicious anaemia, gastric intestinal metaplasia, familial adenomatous polyposis, Lynch syndrome, Peutz-Jeghers syndrome, juvenile polyposis syndrome.

# Tessmer MS, Flaherty KT. AACR Cancer Progress Report 2017: Harnessing Research Discoveries to Save Lives. Clinical cancer research. 2017; 23: 5325. (Consensus among cancer screening recommendations)^53^

⁎ Heaf JG, Hansen A, Laier GH. Quantification of cancer risk in glomerulonephritis. BMC Nephrol. 2018;19: 27. Cancer data extracted from the Danish Renal Biopsy Registry showed that cancer incidence was rare in patients (<45 years) with glomerular diseases but the incidence was increased significantly beyond 45 years of age. This finding suggested that screening should start at the age of 45.^36^

+ Ascherman B, Oh A, Hur C. International cost-effectiveness analysis evaluating endoscopic screening for gastric cancer for populations with low and high risk. Gastric Cancer. 2021: 1-0. This cost-effectiveness analysis suggested that screening for gastric cancer is cost effective in countries with higher incidence of gastric cancer.

Unlike other glomerular diseases, the employment of novel biomarkers can be helpful in distinguishing idiopathic MN from malignancy-related MN. A combination of anti-PLA2R and anti-THSD7A in serum and glomerular PLA2R, THSD7A, and immunoglobulin staining will likely increase diagnostic sensitivity and specificity when screening for underlying malignancies in MN [28]. The utility of NELL-1 as a biomarker for cancer screening in MN requires further study [50,[69], [70], [71]] For cancer screening in other types of glomerular disease, further development in biomarkers {proteins, auto-antibodies, nucleic acids (cell-free DNA and RNA) in serum or other bodily fluids}, with a high degree of sensitivity and specificity are required.

## Conclusion

Neoplasia may not manifest itself at the time of kidney biopsy or soon before/after biopsy proven glomerular disease. It is challenging to discern glomerular disease associated with malignancy from de novo cancer. The increased risk of paraneoplastic tumour is mainly limited to a 6–12-month duration before and after kidney biopsy whereas the risk of de novo cancer increases over time following exposure to immunosuppressants. In patients with glomerular disease, the accurate differentiation between idiopathic and paraneoplastic glomerulopathy is important as these conditions have different therapeutic approaches. Along the disease trajectory of glomerular disease, treating clinicians should maintain a high degree of vigilance with regards to malignancy in the context of disease as early diagnosis is likely to influence treatment decisions which can translate to an improved prognosis. Cancer screening should include comprehensive clinical history and systematic physical examination, extrapolation of existing gold-standard cancer screening guidelines followed by targeted tests utilising glomerular immunoglobulin staining and novel biomarkers which may provide a more precise differentiation between idiopathic and malignancy-associated nephropathy. The development and refinement of reliable biomarkers will improve our insight into the pathophysiology of cancers in patients with glomerular disease and help the differentiation between primary and paraneoplastic glomerulopathy.

## CRediT authorship contribution statement

**Zaw Thet:** Conceptualization, Writing – original draft. **Alfred K. Lam:** Conceptualization, Writing – review & editing. **Dwarakanathan Ranganathan:** Writing – original draft. **Soe Yu Aung:** Writing – original draft. **Thin Han:** Writing – original draft. **Tien K. Khoo:** Conceptualization, Writing – review & editing.

## Declaration of Competing Interest

None.
